# Tissue-Specific Expression of the EWSR1::FLI1 Fusion Protein Identifies *col2a1a*-Positive Cells as a Source of Ewing Sarcoma-like Tumors in Zebrafish

**DOI:** 10.3390/ijms27073131

**Published:** 2026-03-30

**Authors:** Rebecca A. Anderson, Xin Chen, Usua Oyarbide, Nicolas J. Alvarez, Aidan Sievers, Gary K. Schwartz, Seth J. Corey

**Affiliations:** 1 Departments of Pediatrics and Heart, Blood, Kidney Research, Cleveland Clinic Research, Cleveland, OH 44106, USA; rebeccaa24@gmail.com (R.A.A.); chenx11@ccf.org (X.C.); usualab@gmail.com (U.O.); nja54@case.edu (N.J.A.); sievera@ccf.org (A.S.); 2 Case Comprehensive Cancer Center, Cleveland, OH 44106, USA

**Keywords:** Ewing sarcoma, zebrafish, transgenic, developmental biology

## Abstract

Ewing sarcoma (ES) is the second most common primary bone malignancy in children and adolescents and remains one of the most lethal pediatric cancers. Found in more than 85% of patients with ES, *EWSR1::FLI1* results from the t(11;22)(q24;q12) chromosomal translocation. This fusion encodes an aberrant transcription factor that dysregulates gene expression and drives oncogenic transformation. Although this oncogene was identified over three decades ago, therapeutic progress has been limited, in part due to the lack of robust and permissive animal models. Prior efforts to generate transgenic mouse models have been unsuccessful, and while zebrafish have emerged as a promising system, a tissue context capable of supporting EWSR1::FLI1-driven tumorigenesis has not been defined. Here, we report that tissue-specific expression of *EWSR1::FLI1* in zebrafish induces tumor formation that recapitulates the histologic and molecular hallmarks of human ES, including small round blue cell morphology and characteristic biomarker expression. Tumors were driven by the *col2a1a* promoter and resulted in ~70% incidence of notochord tumors within the first 72–96 h. Of the surviving fish, ~5% developed CD99-positive small round blue cell tumors at ~9 months post-fertilization. This work establishes a stable tissue-specific transgenic model of ES, providing a powerful in vivo platform to investigate disease pathogenesis and evaluate novel therapeutic strategies.

## 1. Introduction

Ewing sarcoma (ES) is the second most common bone cancer in children, and one of the most lethal pediatric cancers. Approximately 250 individuals in the US are annually diagnosed with ES [1]. Overall five-year survival with limited disease is 60%, and those with recurrent or relapsed disease is less than 20% [2]. Nearly 25% of patients have metastatic disease at time of diagnosis [3,4]. Current treatment includes an intensive regimen of five chemotherapeutic agents with a high level of toxicity [5]. Recurrent ES is currently incurable.

**Figure 1:**
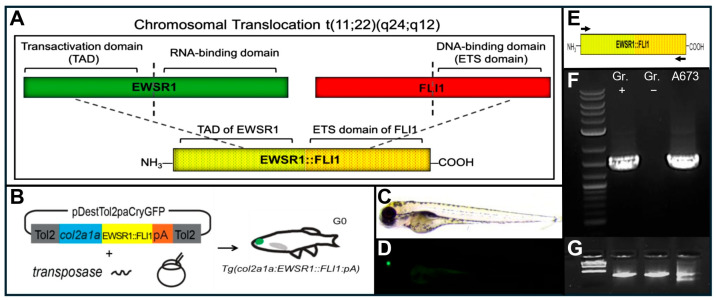
Generation and validation of transgenic *EWSR1::FLI1* zebrafish. (**A**) Schematic of ES translocation t(11;22)(q24;12) in EWSR1::FLI1 fusion showing the N-terminal domain of EWSR1, which contains transactivation domain (TAD), and its fusing with the C-terminal DNA-binding domain of the ETS transcription factor FLI1. (**B**) The *col2a1a* promoter is used to drive expression of the human EWSR1::FLI1 fusion construct in transgenic zebrafish created using the Tol2 transposase method. (**C**,**D**) Identification of transgene-positive larvae by GFP expression in the lens driven by the *cryaa*:EGFP cassette at 2 days post-fertilization (dpf). (**E**) Schematic showing location of human-specific primer set used to amplify fusion construct from RNA using semi-quantitative RT-PCR. (**F**) Detection of the 1502 bp *EWSR1::FLI1* fusion transcript in transgene-positive larvae and ES cell line A673. (**G**) β-actin was used as the internal control for qRT-PCR. Gr+, green eye positive. Gr−, green eye negative.

Nearly 90% of ES cases harbor the characteristic chromosomal translocation t(11;22)(q24;q12), which generates a fusion between the *Ewing sarcoma breakpoint region 1* (EWSR1) gene and the *Friend leukemia virus integration 1* (FLI1) gene (Figure 1A) [6,7]. EWSR1, a member of the TET family of RNA-binding proteins, is frequently involved in chromosomal translocations that fuse its N-terminal transactivation domain to the DNA-binding domains of various ETS family transcription factors, such as FLI1. The resulting EWSR1–ETS fusion proteins act as aberrant transcriptional regulators that drive oncogenic transformation in ES and related sarcomas. This leads to a ubiquitously expressed fusion protein that is believed to act as an aberrant transcription factor, promoting transformation through dysregulated gene expression and leading to cancer within bone and soft tissue [8,9].

ES is most often associated with the bone, with 40% of cases arising in the leg; approximately 15–20% of ES cases are extraosseous, with the paravertebral region being the most common site [10]. A histologic feature of all ES tumors is a small round blue cell tumor (SRBCT) morphology. Hematoxylin and eosin (H&E) staining of tumors show undifferentiated cells with little cytoplasm and small, round nuclei that stain deep blue [11]. A common characteristic of ES tumors, although not specific to ES, is diffuse membranous staining by the cell-surface glycoprotein CD99 [11]. ES tumors also typically stain positive for periodic acid Schiff (PAS) due to a high cytosolic glycogen content [11].

Despite the molecular identification of the oncogenic transcription factor in ES in 1993, there has been little progress in developing better therapies [12]. A major obstacle to developing new, more effective therapies against ES is the lack of animal models [5]. Prior attempts at producing a transgenic model of ES in the mouse have proved unsuccessful [13]. Leacock et al. [14] and Vasileva et al. [15] have shown the zebrafish to be a promising model to study ES, yet they have not identified a cell group permissible to EWSR1::FLI1 expression. Here, we report that tissue-specific expression of *EWSR1:FLI1* drives the development of tumors, which recapitulate the characteristics of ES. This is the first reported tissue-specific stable transgenic model of ES.

## 2. Results

### 2.1. col2a1a-Expressing Cells Act as a Permissive Environment for EWSR1::FLI

To overcome early developmental toxicity associated with ubiquitous EWSR1::FLI1 expression, we selected the *col2a1a* promoter to drive expression of the fusion oncoprotein in the zebrafish. (The human ortholog is *COL2A1*.) Expression of *col2a1* occurs in chondrocytes and is conserved from teleost (bony fish) to mammals [16,17,18]. In the zebrafish, *col2a1a* expression is first detected in the notochord at 10 h post-fertilization (hpf) [18,19]. Similarly to human development, the notochord in zebrafish is derived from axial mesodermal cells that undergo differentiation to form large vacuolated epithelial cells [20]. In both zebrafish and humans, the embryonic notochord organizes the development of the axial skeleton. Most bones in the human body are endochondral, i.e., they develop from a cartilage template. Consistent with human bone development, expression of *col2a1a* in the zebrafish is found within the resting and proliferating chondrocytes of the endochondral growth plates [19,21].

To compare the effects of tissue-specific EWSR1::FLI1 expression to that of ubiquitous expression, we injected the constructs *Tg(col2a1a:EWSR1::FLI1:pA*) (Figure 1B) and *Tg(ubi:EWSR1::FLI1:pA*) into wild-type zebrafish at the one-cell stage. The transgene backbone of each construct contains the *cryaa*:EGFP element, allowing for the identification of transgene-positive fish at 2 dpf by green lens fluorescence (Figure 1C,D). Approximately 97% of the *Tg(ubi:EWSR1::FLI1:pA*) larvae (292/300 injected embryos) were dead or grossly malformed by 3 dpf, indicating ubiquitous expression of EWSR1:FLI1 results in embryonic lethality [14,15]. Conversely, nearly all the *Tg(col2a1a:EWSR1::FLI1:pA*) injected embryos were alive at 3 dpf and showed no gross congenital malformations. To confirm transgene expression of the oncoprotein in viable *Tg(col2a1a:EWSR1::FLI1:pA)* larvae, we extracted RNA from green-eyed transgene-positive fish at 7 dpf and used semi-quantitative RT-PCR and human-specific primers (Table 1) to amplify the fusion construct (Figure 1E). Expression of *EWSR1::*FLI1 was detected in green-eyed larvae but not in non-green-eyed siblings with actin as a control (Figure 1F,G). Thus, selective tissue expression of *EWSR1::FLI1* by *col2a1a* does not result in embryonic lethality, and *col2a1a* expression domains provide a permissive environment for the expression of the *EWSR1::FLI1* oncoprotein.

### 2.2. Expression of EWSR1::FLI1 Within Cartilaginous Elements Results in DNA Damage and Craniofacial Defects During Early Development

**Figure 2:**
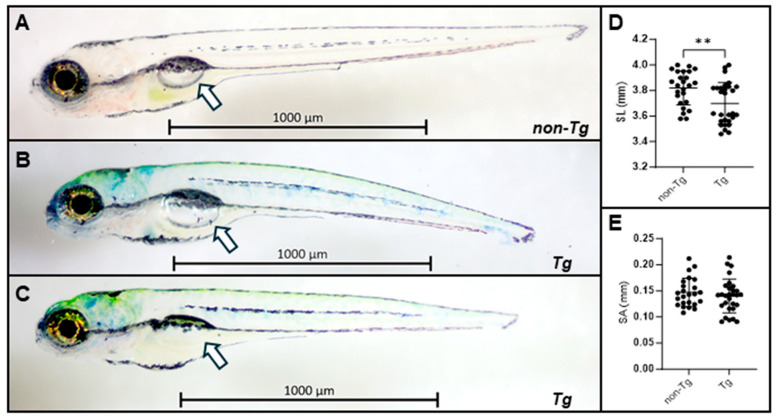
Morphologic characterization of transgenic EWSR1::FLI1 zebrafish. (**A**) Phenotypic characterization of non-transgenic siblings at 6 dpf. (**B**,**C**) Phenotypic characterization of Tg(*col2a1a:EWSR1FLI1:pA*) at 6 dpf. The white arrows indicate the swim bladder. The bubble characterizing the seim bladder is greatly diminished in panel (**C**). (**D**) Quantification of standard length (SL) in transgenic (*n* = 29) and non-transgenic (*n* = 25) larvae at 6 dpf. For non-transgenic fish, the SL is 3.82 ± 0.13 mm, and 3.70 ± 0.16 mm for transgenic fish; ** *p* < 0.01 by unpaired Student’s *t*-test. (**E**) Quantification of surface area (SA) of swim bladder in transgenic (*n* = 29) larvae with swim bladder and non-transgenic (*n* = 24) larvae at 6 dpf. Only living fish for each condition were studied. For non-transgenic fish, the SA is 0.147 ± 0.027 mm, and 0.143 ± 0.03 for transgenic fish; *p* = 0.423. The *EWSR1::FLI1* transgenic fish were smaller in standard length. Because the immature swim bladders did not statistically differ between the lines at 6 dpf and the gross morphology showed no difference, we conclude that the smaller standard length was not due to a developmental factor.

While the *Tg(col2a1a:EWSR1::FLI1:pA)* larvae showed no gross deformities during the first seven days of development, they displayed morphological phenotypes (Figure 2 and Supplemental Figure S1). Beginning around 3 dpf, some fish displayed enlarged ears, affecting them unilaterally (Figure S1A) or bilaterally (Figure S1B). Although the ears were enlarged, patterning of the ear was not grossly abnormal. Craniofacial defects were highly penetrant, with most G0 fish showing some degree of malformation by 7 dpf (Figure 2B,C). Notably, close to 60% of transgenic fish did not develop a swim bladder by 7 dpf (Figure 2A,B versus Figure 2C). The fish that did not develop swim bladders by 7 dpf died. The lack of swim bladder development did not directly correlate with the presence of ear defects and/or craniofacial defects. Overall, transgenic fish were smaller than their non-transgenic siblings (Figure 2D). When comparing the transgenic fish that had swim bladders to the non-transgenic fish, the transgenic fish that developed swim bladders displayed no difference in swim bladder size (Figure 2E), indicating that their decreased size was unlikely due to a developmental delay.

To characterize the craniofacial skeletal defects, we used Alcian Blue to stain the craniofacial cartilage at 6 dpf (Figure S1C–H). Comparison of transgenic fish to non-transgenic siblings revealed patterning defects in the cartilaginous elements of the transgenic larvae. While there were no obvious fusions or deletions of elements, the angels of the ceratohyal (ch) and Meckel’s cartilage (mc) were noticeably narrowed (Figure S1E,H). The ethmoid plate (ep) appeared shortened (Figure S1F,G), with a severe shortening observed in mc (Figure S1G,H).

**Figure 3:**
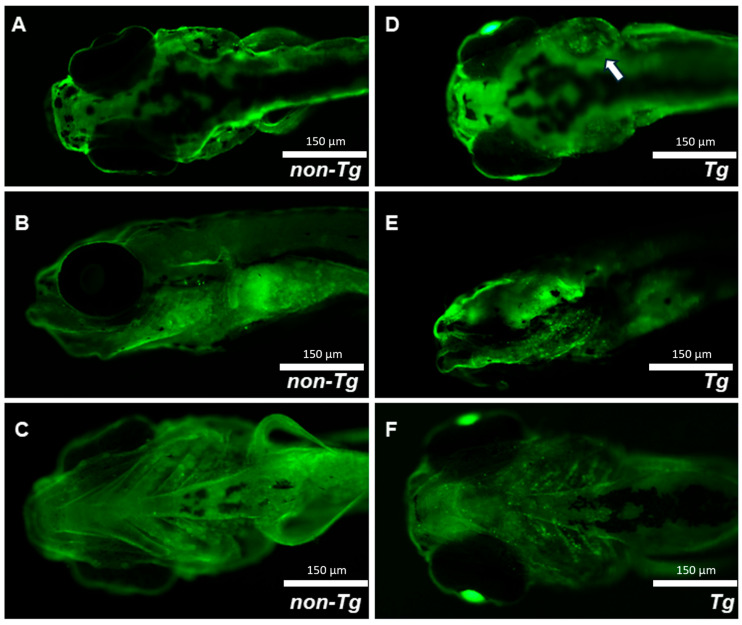
Increased DNA double-strand breaks in transgenic EWSR1::FLI1 zebrafish. γ-H2AX staining in craniofacial structures with the following: (**A**) Non-Tg dorsal view. (**B**) Non-Tg lateral view. (**C**) Non-Tg ventral view. (**D**) Tg dorsal view, with the white arrow identifying abnormal ear formation. (**E**) Tg lateral view. (**F**) Tg ventral view. These findings suggest that aberrant transcriptional activity driven by EWSR1::FLI1 induces genomic stress with R-loops and DNA double-strand breaks [22] during development, as reflected by increased γ-H2AX staining.

To investigate how expression of the EWSR1::FLI1 fusion protein in chondrocytes might cause severe compression or shortening of skeletal elements and associated cartilage defects, we examined DNA damage in craniofacial cartilage using the γ-H2AX antibody at 7 dpf (Figure 3A–F). Transgenic larvae exhibited a pronounced increase in DNA double-strand breaks within the cartilage, as evidenced by markedly elevated γ-H2AX staining in craniofacial structures such as the ep (Figure 3D), ch (Figure 3E,F), hyosymplectic (hs) (Figure 3E), ceratobranchials (cb), and mc (Figure 3F). Furthermore, γ-H2AX was also observed in the enlarged ear(s) of transgenic larvae (Figure 3D). These results indicate that expression of the fusion protein within cartilaginous elements during early development results in DNA damage and subtle patterning defects.

### 2.3. Tg(col2a1a:EWSR1::FLI1:pA) Larvae Develop Notochordal Neoplasms During Early Development

**Figure 4:**
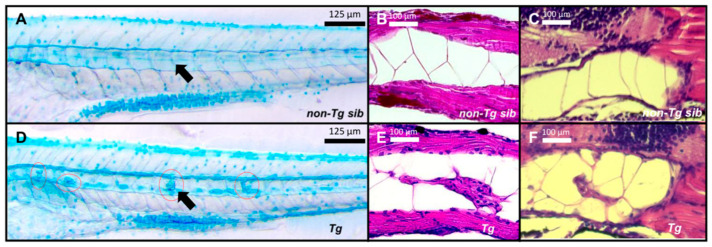
Development of notochord tumors in zebrafish. (**A**) Alcian Blue staining of non-Tg larvae at 6 dpf. (**B**) Coronal section of non-Tg larvae tail at 6 dpf with H&E staining. (**C**) Sagittal section of non-Tg larvae tail at 6 dpf with H&E staining. (**D**) Alcian Blue staining of Tg larvae at 6 dpf. The red circles denote notochordal neoplasms. Black arrows indicate the notochord in panels (**A**,**D**). (**E**) Coronal section of Tg larvae tail at 6 dpf with H&E staining. (**F**) Sagittal sections of Tg larvae tail at 6 dpf with H&E staining. These data show that expression of *EWSR1::FLI1* in *col2a1a*-expressing cells disrupts normal notochord organization and induces dysplastic notochordal growths during early zebrafish development, as demonstrated by abnormal cellular proliferation and proteoglycan-rich lesions detected by Alcian Blue staining and histological analysis.

In addition to the cartilaginous elements of the skeleton, expression of *col2a1a* is also found within the notochord. In zebrafish, the detection of *col2a1a* is first seen within the notochord and strong notochordal expression continues throughout early development [19,23]. Intriguingly, the *Tg(col2a1a:EWSR1::FLI1:pA)* larvae developed multifocal notochordal growths detectable by microscopic examination (Figure 4A,D–F). Notochord growths represented a highly penetrant phenotype, with ~70% of transgenic larvae exhibiting notochord dysplasia by 5 dpf. The occurrence of notochord defects did not directly correlate with the absence of swim bladder inflation. To further characterize these lesions, larvae were stained with Alcian Blue, which labels negatively charged proteoglycans containing sulfated and/or carboxylated glycosaminoglycans (GAGs) such as heparan sulfate, chondroitin sulfate, and hyaluronic acid. The notochord growths showed intense Alcian Blue staining and were readily visible within the notochord (Figure 4A,F). The extracellular matrix of the notochord growths is enriched in negatively charged proteoglycans. To further examine the notochord architecture and the nature of the growths, we performed hematoxylin and eosin (H&E) staining on coronal (Figure 4B) and sagittal (Figure 4C) sections of 6 dpf larvae. In non-transgenic siblings, the notochord exhibited the typical organization of large vacuolated cells (Figure 4B). In contrast, transgenic larvae displayed focal regions of cellular hyperplasia, characterized by clusters of cells within a dysplastic notochordal structure (Figure 4C). No spatial bias toward either the anterior or posterior notochord was observed. While the growths appeared dysplastic, they did not resemble the SBRC morphology associated with ES.

### 2.4. Development and Characterization of ES-like Tumors Driven by EWSR1::FLI1 Fusion Protein

**Figure 5:**
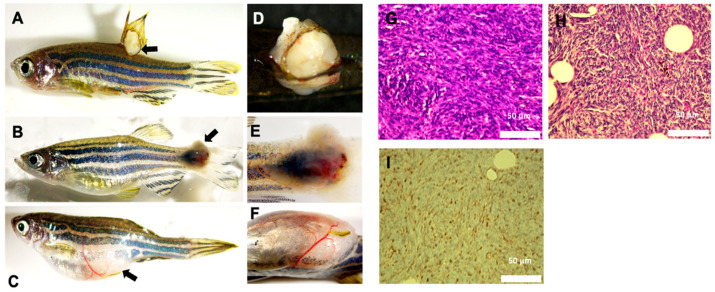
Characterization of ES-like tumors in adult zebrafish. (**A**) Lateral view of 15-month-old transgenic fish with tumor on dorsal fin. (**B**) Lateral view of 10-month-old transgenic fish with mass on caudal fin. (**C**) Lateral view of 12-month-old transgenic fish with mass near anal fin. Black arrows indicate ES-like tumors in (**A**–**C**). (**D**) Magnified image of dorsal fin tumor, dorsal view. (**E**) Magnified image of caudal fin tumor, lateral view. (**F**) Magnified image of anal fin tumor, ventral view. (**G**) Histological analysis of the zebrafish anal fin tumor with H&E staining. (**H**) PAS staining of the zebrafish anal fin. (**I**) Histological analysis of the zebrafish anal fin tumor upon CD99 staining, which was performed in two independent fish tumors. These findings indicate that a subset of adult Tg(*col2a1a:EWSR1::FLI1:pA*) zebrafish develop tumors with histological and immunophenotypic features consistent with ES neoplasms, including small round blue cell morphology on H&E staining, glycogen accumulation detected by PAS staining, and expression of the diagnostic cell-surface marker CD99.

While the Tg(col2a1a:EWSR1::FLI1:pA) are fully viable during the first 7 dpf, only ~40% of transgenic fish were able to survive past 7 dpf due to defective swim bladder development (Figure 2C). Of the fish that survived past 7 dpf, approximately 5% (9 out of 185 adult fish) went on to develop grayish-white, fleshy, vascularized tumors near and around the bony tissues of the fins in adulthood (Figure 5A–F). ES often presents as a fleshy, gray-white, lobulated tumor with a somewhat soft surface [24]. Phenotypically, the tumors observed in adult Tg(col2a1a: EWSR1::FLI1:pA) zebrafish closely resembled those of human ES. To determine if the Tg(col2a1a: EWSR1::FLI1:pA) tumors met the histological criteria of ES, we performed H&E, PAS, and CD99 staining on paraffin sections of the zebrafish tumors. Histological analysis of the zebrafish tumors revealed a SRBC morphology upon H&E staining (Figure 5G), a positive PAS staining (Figure 5H), and positive CD99 staining (Figure 5I). These results indicate that the tumors that develop in adult Tg(col2a1a: EWSR1::FLI1:pA) zebrafish mimic those of ES. This suggests that expression of the fusion oncoprotein within col2a1a-expressing cells allows for the development of ES.

**Figure 6:**
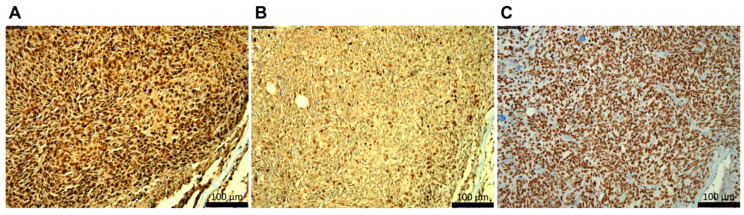
Development and characterization of ES-like tumors in adult zebrafish. Histological analysis of the zebrafish anal fin tumor upon (**A**) PCNA, (**B**) phospho-γH2AX, and (**C**) Sox10 staining. Altogether, these transgenic *EWSR1::FLI1* zebrafish-driven, ES-like tumors are proliferative, display DNA damage, and possess a marker for neural crest cells.

Immunohistochemical staining of the EWSR1::FLI1-driven tumors showed intense staining for the proliferating cell nuclear antigen (PCNA) marker with less intense staining for phospho-γH2AX (Figure 6A,B). Based on the development of notochord tumors, we speculated that adult ES-like tumors might be related to neural crest cells. In support of this, strong staining for the transcription factor Sox10, a sensitive marker for neural crest stem cell, was detected in the tumors (Figure 6C) [25]. Further work, such as scRNA-Seq, needs to be done to confirm this.

## 3. Discussion

Neither genetically engineered mouse models nor mammalian cell lines of ES generated through ectopic expression of the EWSR1::FLI1 fusion oncogene have been reliably established. This difficulty is widely attributed to the inherent toxicity of the fusion protein [13] and has hindered progress in developing new therapeutic strategies. The low tumor mutational burden and lack of additional genetic lesions at diagnosis support the notion that the fusion protein alone is sufficient to drive the development of ES [9]. Here, we report the establishment of a stable transgenic zebrafish strain, which expresses the human *EWSR1::FLI1* fusion that developed multifocal notochord tumors within ~3 dpf and SRBCT encasing bone at ~9 mpf. The SRBCT stained positively for CD99, which is almost always expressed on human ES cells. Our work is novel in that it demonstrates the following: (1) *col2a1a* cells allow expression of the EWSR1::FLI1 and do not die; (2) expression in these cells leads to ES-like tumor formation.

We believe that the SRBCT emanate from a skeletal-associated lineage. This is supported by several observations: (1) The tumors arose in close proximity to regions of active bone growth. (2) As shown in Figure 5D, the tumor is wrapped around the bone. The skeletal rays (fins) of the fish are dermal bones. Directly below the rays are the distal and proximal radials (endoskeletal supports). This location is where the tumors are developing. (3) As shown Figure 3, increased DNA damage is accompanied by disruption of growth plate organization.

We first used the *ubi* promoter, which failed to produce viable zebrafish. Choosing to use the *col2a1a* promoter that is active in embryonic skeletal, neural crest, and notochord tissues [19], we were successful in generating transgenic fish that survived into adulthood. The EWSR1::FLI1 transgenic larvae demonstrated a high penetrance of multifocal notochord tumors beginning around 3 dpf. The lack of viability with the ubiquitous promoter suggests that the oncofusion protein is broadly toxic to the organism, although certain tissues appear able to tolerate its expression. The fate of these notochord tumors remains unclear, as adult zebrafish show neither persistent notochord abnormalities nor evidence of chordomas [23]. Moreover, the notochord tumors do not support a neural crest cell of origin as the notochord is not derived from neural crest cells. Instead, the neural crest cells migrate around the notochord to form neurons and glia [26].

There has been ongoing debate regarding the cell of origin for ES, with mesenchymal and neural crest lineages being favored [27,28]. The ear and notochordal defects seen in our transgenic fish are likely due to the strong expression of *col2a1a* in the otic vesicle and notochord [19] driving the oncofusion protein expression. The craniofacial defects are likely a result of *col2a1a* expression, driving EWSR1::FLI1 expression in the proliferating chondrocytes of the growth plates in the endochondral bones of the craniofacial skeleton. These results demonstrate that embryonic osteochondrogenic progenitors are a permissive environment for expression of the EWSR1::FLI1 oncoprotein. The development of ES-like tumors in our transgenic zebrafish model affirms that embryonic osteochondrogenic progenitors are permissive to ES [29]. Furthermore, multiple lines of evidence suggest that ES may arise from chondrocyte as followss: (1) expression of chondrocyte-associated genes such as *SOX9* and *COL2A1*, (2) the ability of *EWSR1::FLI1* to direct mesenchymal stem cells toward a chondrogenic program, and (3) the frequent occurrence of ES near growth plate regions, where chondrocytes reside. Col2a1a is a strong marker for resting and proliferating chondrocytes in the growth plate, with expression decreasing as cells undergo hypertrophic differentiation and becoming minimal in late-stage chondrocytes.

Using a different approach to develop ES tumors in zebrafish, Vasileva et al. showed that EWSR1::FLI1 can reprogram neural crest cells toward a mesenchymal state [30]. In this conditional transgenic zebrafish model, embryonic injection of a *Cre* recombinase, *Tol2* transposase, and *ubi*-driven *EWSR1::FLI1* produced SRBCT [15,30]. To demonstrate involvement of neural crest cell progenitors, the conditional expression was demonstrated by using the murine *Sox10* promoter. Thus, zebrafish modeling can provide insights into which tissues can tolerate the EWSR1::FLI1 protein, but such insights would be tempered by the specific promoter. The cell of origin has been debated for some time, with sides favoring either neural crest or mesenchymal stem cell. Because of the Sox10 staining (Figure 6C), we cannot rule out a neural crest cell. However, SOX10 also marks differentiating chondrocytes in the growth plate and articular cartilage [31]. Altogether, the data presented here on our *col2a1a*-driven *EWSR1::FLI1* transgenic model favors more a mesenchymal stem cell or chondrocyte/cartilage cell of origin. More definitive studies aiming to identify the ES cell of origin are proceeding with lineage tracing experiments, time-lapse imaging of the growth plates/tumors, and double in situ hybridization on the growth plates/tumors.

In transgenic zebrafish, the integration site and copy number of a transgene are not routinely determined because the construct can integrate essentially anywhere in the genome and may do so multiple times. As a result, each G0 fish may carry the transgene at a different genomic insertion site. In this study, we are not comparing this transgenic line to other lines. Rather, we report two observations: (1) cells expressing *col2a1a* permit expression of the fusion protein, and (2) expression of the fusion protein within *col2a1a*-expressing cells leads to tumor formation. Besides the use of promoters other than *col2a1a* that might drive tumor formation in other tissues, limitations to our model include the long latency period and low penetrance. ES arises with a median age of 15 years. Zebrafish typically live for two years, and ES-like tumors did not develop until ~9 mpf. Thus, aging may play a role in carcinogenesis. Although the penetrance of ES-like tumors in the transgenic zebrafish is low, ES is also a rare cancer in humans, with only ~1000 cases diagnosed annually throughout the world.

No molecularly targeted therapy currently exists for ES [32]. Conditional or stable transgenic zebrafish lines can be used to screen potential drugs to target EWSR1::FLI1 or its downstream targets. Because zebrafish acquire components of the adaptive immune response within 4–6 weeks post-fertilization [33,34,35], these organismal models can also be used to evaluate the efficacy of immunotherapy.

## 4. Materials and Methods

### 4.1. Creation of Transgenic Lines

The cDNA sequence of human EWSR1::FLI1 type 1 fusion was amplified from the pCDH-puro-EWSR1-FLI1 plasmid (Addgene #102813, Watertown, MA, USA) [36] using *att*B PCR primers (Table 1) and cloned using the Gateway BP reaction on the pDONOR221 plasmid. To create the *Tg(ubi:EWSR1::FLI1:pA)* and the Tg*(col2a1a:EWSR1::FLI1:pA)* lines, the cDNA sequence of EWSR1::FLI1 was cloned between the *ubi* and *col2a1a* promoters, respectively, and a polyA sequence in the pDestTol2pACryGFP vector was cloned using Gateway LR reactions. The constructs were confirmed by restriction enzyme analysis and sequencing. The *col2a1a* promoter on the p5E plasmid was a kind gift from Dr. Rodney Dale (Loyola University, Chicago, IL, USA). The other plasmids are available in the Tol2kit (Addgene, Watertown, MA, USA) [37]. The transgenic lines were created using the Tol2 transposase method [37]. Transgene-positive fish were identified at 2 days post-fertilization (dpf) by GFP-positive lens and PCR genotyping with genome DNA.

### 4.2. Semi-Quantitative RT-PCR

Twenty *Tg(col2a1a:EWSR1::Fli1:pA)* larvae with green eyes, as well as twenty non-transgenic siblings without green eyes, were collected at 7 dpf. The fish were pooled, and RNA was extracted using Trizol (ThermoFisher Scientific, Waltham, MA, USA). Using 175 ng RNA from each pool and primers specific to human EWSR1 and FLI1 (Table 1), PCR was performed using the Superscript III One-Step RT-PCR System with Platinum Taq DNA Polymerase (ThermoFisher Scientific, Waltham, MA, USA). The PCR products were separated using 3.5% Metaphor agarose and 1% agarose gels.

### 4.3. Alcian Blue Staining

Larval fish were euthanized and fixed overnight in phosphate-buffered 10% formalin. Cartilage staining was performed as previously described [38].

### 4.4. Histological Sections

Larvae and adult fish were euthanized and fixed overnight in phosphate-buffered 10% formalin. Adult fish were treated for eight hours with 0.5M EDTA prior to processing. Sectioning, H&E staining, and antibody staining were carried out in the Cleveland Clinic Histology Core. Slides were incubated with anti-CD99 antibody (ab108297, Abcam at 1:100, Cambridge, UK), phospho-γH2AX (GTX 127342, GeneTex at 1:1000, Irvine, CA, USA), PCNA (GTX 124496, GeneTex at 1:1000, Irvine, CA, USA), and Sox10 (GTX 128374, GeneTex at 1:250, Irvine, CA, USA). All images were taken using ZEISS (Oberkochen, Germany) stereoscopes (Stemi 508 and Discovery V8) and an AxioImager M2 microscope equipped with a camera (Axiocam, Oberkochen, Germany). H&E and PAS staining were performed using commercially available kits in the histology core at Cleveland Clinic.

### 4.5. Zebrafish Lines and Maintenance

The following wild-type and transgenic fish lines were used: AB (ZFIN ID: ZDB-BENO-960809-7), *Tg(ubi:EWSR1::FLI1:pA)*, and *Tg(col2a1a:EWSR1::FLI1:pA)*.

### 4.6. Statistical Analysis

Unpaired Student’s *t*-test was performed using GraphPad Prism Version 10.6.1.

## 5. Conclusions

A stable zebrafish line expressing the human *EWSR1::FLI1* fusion gene under the control of the *col2a1a* promoter survives past adulthood, suggesting that chondrocytes represent a permissive cellular context for *EWSR1::FLI1* expression. Bone-associated SRBCT form at around 9 mpf. These tumors express markers of human ES, such as CD99 and SOX10. We are currently breeding them with other zebrafish lines to accelerate tumor formation. Our transgenic zebrafish model can provide an organismal model to test direct or indirect targets of the oncofusion protein.

## Figures and Tables

**Table 1:** Primer sequences.

attB_EWSR1::FLI_F	GGGGACAAGTTTGTACAAAAAAGCAGGCTCACCATGGCGTCCACGGATTACAGTAC
attB_EWSR1:FLI_R	GGGGACCACTTTGTACAAGAAAGCTGGGTCCTAGTAGTAGCTGCCTAAGTGTG
hEWSR1::FLI1_F	CACCATGGCGTCCACGGATTACAG
hEWSR1::FLI1_R	CCTAGTAGTAGCTGCCTAAGTGTG
beta-actin_F	TGCTGTTTTCCCCTCCATTG
beta-actin_R	TTCTGTCCCATGCCAACCA

## Data Availability

The original contributions presented in this study are included in the article/Supplementary Materials. Further inquiries can be directed to the corresponding author(s).

## Supplementary Materials

The following supporting information can be downloaded at https://www.mdpi.com/article/10.3390/ijms27073131/s1.

## Abbreviations

The following abbreviations are used in this manuscript:

bp	base pair
ch	ceratohyal
dpf	days post-fertilization
ep	ethmoid plate
ES	Ewing sarcoma
EWSR1	Ewing sarcoma breakpoint region 1
FLI1	Friend leukemia virus integration 1
G0	generation 0
H&E	hematoxylin and eosin
h	hour
hpf	hours post-fertilization
mc	Meckel’s cartilage
mpf	months post-fertilization
pA	polyA
PAS	periodic acid Schiff
PCNA	proliferating cell nuclear antigen
qRT-PCR	quantitative reverse transcription polymerase chain reaction
SRBCT	small round blue cell tumor
Tg	transgenic
ubi	ubiquitin
