# Supplementary material for: Tissue-Specific Expression of the EWSR1::FLI1 Fusion Protein Identifies col2a1a-Positive Cells as a Source of Ewing Sarcoma-like Tumors in Zebrafish
Source: Int J Mol Sci. 2026 Mar 30;27(7):3131. doi: 10.3390/ijms27073131 (PMC13073343; doi:10.3390/ijms27073131)
Supplement: Supplementary file 1 [file ijms-27-03131-s001.zip › ijms-4187046-supplementary.pdf]

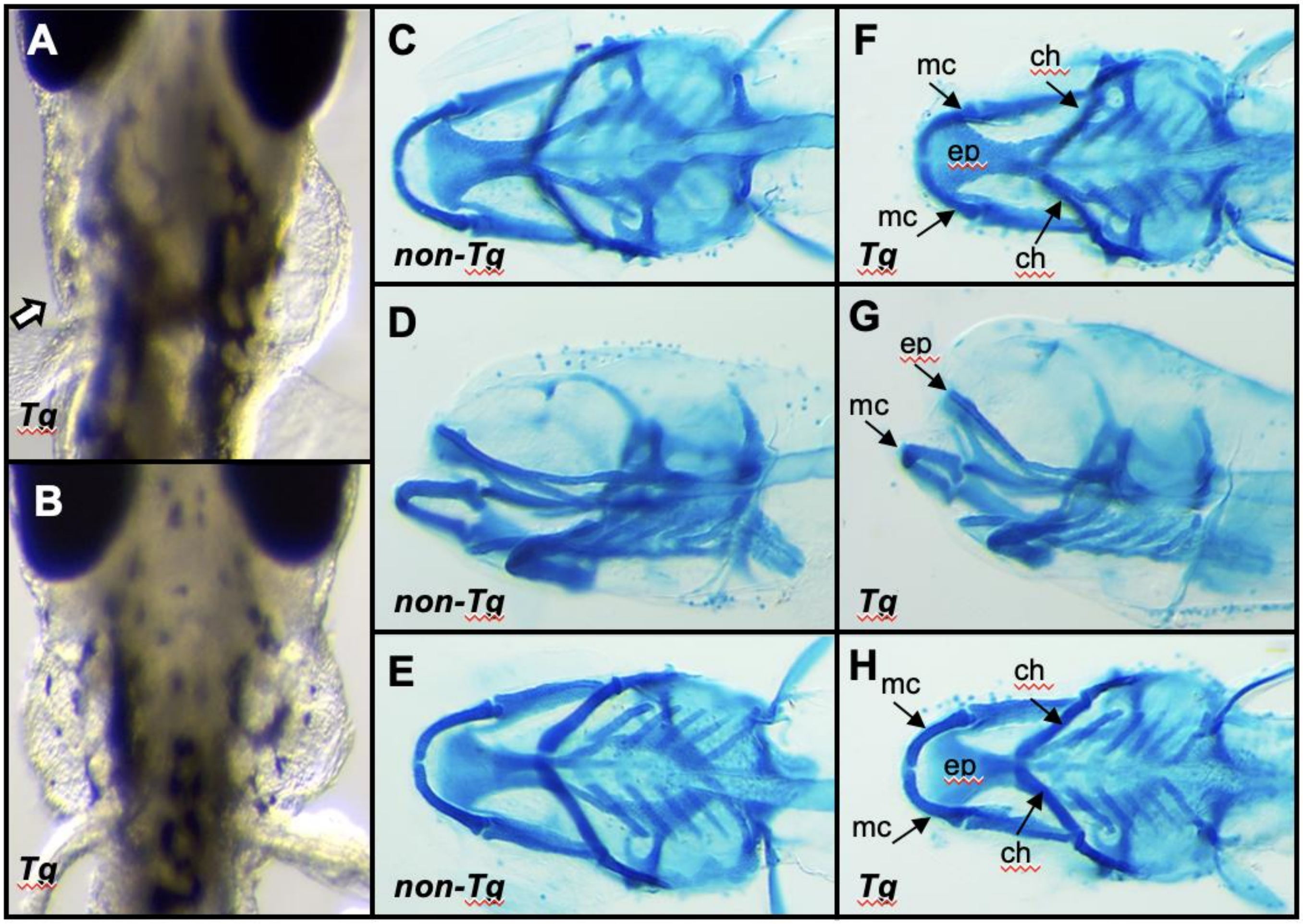

Supplementary Figure S1. Cartilaginous anomalies during zebrafish development. (A) Phenotypic characterization of Tg(*col2a1a:EWSR1::FLI1:pA*) gross morphology at 6 dpf reveals ear defects affecting one ear or (B) both ears. White arrows indicate a normal ear. The lump on the other side and the bilateral lumps in panel (B) are swollen ear anomalies. (F,G,H) Alcian Blue staining of transgenic craniofacial cartilage shows craniofacial morphogenesis defects compared to (C,D,E) non-transgenic craniofacial cartilage. (A,B,C,F) Dorsal view, (D,G) lateral view, and (E,H) ventral view. Tg, transgenic. Non-Tg, non-transgenic sibling.
